# 3D didactic model and useful guide of the semicircular conducts

**DOI:** 10.1590/S1808-86942011000300006

**Published:** 2015-10-19

**Authors:** Ricardo D'Albora Rivas

**Affiliations:** 1 Associate Professor. Vestibular Departement. Clinicas Hospital. School of Medicine of Uruguay. University of the Oriental Republic of Uruguay

**Keywords:** vestibule labyrinth, semicircular canals, vestibular diseases

## Abstract

Knowledge of the anatomy and physiology of the semicircular canals and their central pathways is essential for the diagnosis of vestibular pathology. This 3 dimensional (3D) scheme of the Semicircular Canals (SSCC) is a teaching tool and a useful reference guide for rapid consultation.

**Material and methods:**

A multicolored cardboard model is accompanied by a user manual which provides a thorough description of the tool for the most common vestibular diseases.

**Results:**

Although results cannot be quantitatively assessed, the model has been well received at several Latin American scientific conferences. The model is often understood with verbal instruction only; nevertheless, a printed user manual is included.

**Conclusions:**

This 3 dimensional (3D) model of the Semicircular Canals (SSCC) is a practical, low cost tool for use in private and academic settings.

## INTRODUCTION

The management of vestibular pathology, peripheral or central, requires prior knowledge of the anatomy and physiology^1^, ^2^, ^3^, ^4^, ^5^, ^6^ of the semicircular canals^2^, ^3^, ^5^ and their central pathways 7. The anatomy and pathology of the semicircular canals are particularly difficult to interpret due to the complex geometry and physiological variations between the horizontal and the vertical semicircular canals.

This 3D model of the Semicircular Canals (SSCC) is intended for use as a didactic tool and a quick reference guide when dealing with various pathologies of the labyrinth. It is especially effective in the diagnosis and possible treatment of Benign Paroxysmal Positional Vertigo (BPPV)^8^, ^9^, ^10^, ^11^, as well as the interpretation of the causes of Vestibular Hypofunction^12^, per the Head Thrust Test^7^, ^9^ and in the identification of different Neuritis (inflammatory neuropathies), Canal Fistulas^9^ and Vascular Disorders, based on a knowledge of physiology, innervation and vascular supply of the SSCC 4. It also describes Downbeat and Upbeat Nystagmus (Pitch) and Torsional Nystagmus (Roll)^7^ as a part of the Central Vestibular Pathway in a simple and practical way. It is not intended for use as an anatomically correct model, but as a rough spatial reference. The model does not intend to explain the clinical examination nor pathology in detail, however, and requires some baseline knowledge of the vestibular system for a more complete understanding.

**It is not intended to replace more complete reference books in this area. The model (SSCC3D) is portable and easy to use**.

## MATERIAL AND METHODS

The multicolored cardboard model (Table 1) is accompanied by a 22-page user manual which provides a thorough description of the tool in the setting of the most common examples of vestibular pathologies.

**Table 1:** Messures of the model

FOLDED	cm	INCH
Height	7	2.75
Lenght	14	5.5
UNFOLDED	cm	INCH
Height	7	2.75
Lenght	19.6	7.5
Depht	9.8	3.75
Weight	9gr	

## RESULTS

**Figure 1:**
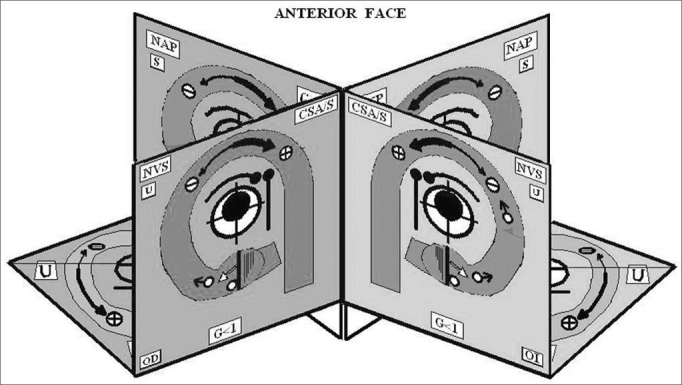
Anterior Face

**Figure 2:**
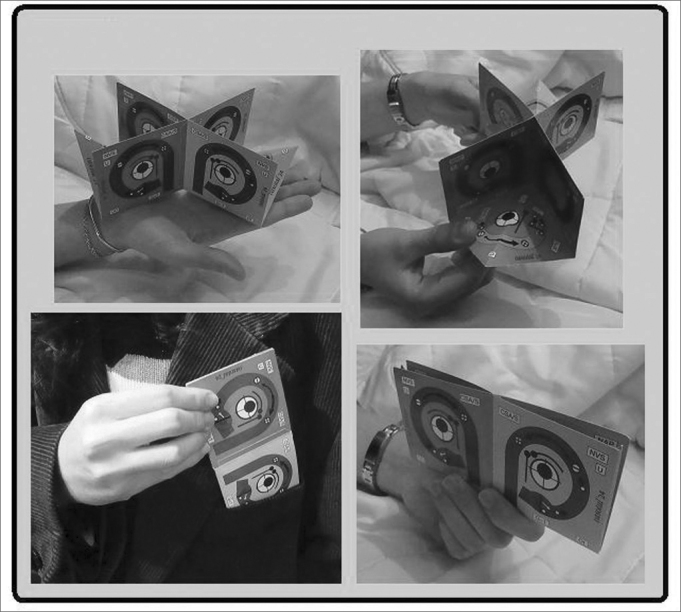
3D Model

This 3D Model is the result of amalgamation of the anatomy and physiology of the semicircular canals. In the model, both labyrinths are shown next to each other, the right to the left, for practical purposes. The left labyrinth is presented on a light blue background and the right labyrinth on a pink background. The model has 12 faces and the SSCC are represented on 10 of the faces in the three axes of space, front to back, with their corresponding ampullas and crista ampullaris. Each SSCC is color coded, and those which share the same color work together when stimulated. The Right Anterior Semicircular Canal and the Left Posterior Semicircular Canal are in blue. The Right Posterior Semicircular Canal and the Left Anterior Semicircular Canal are in green. Both Semicircular Horizontal Canals are in grey (Fig. 1) (Fig. 2)

**Figure 3:**
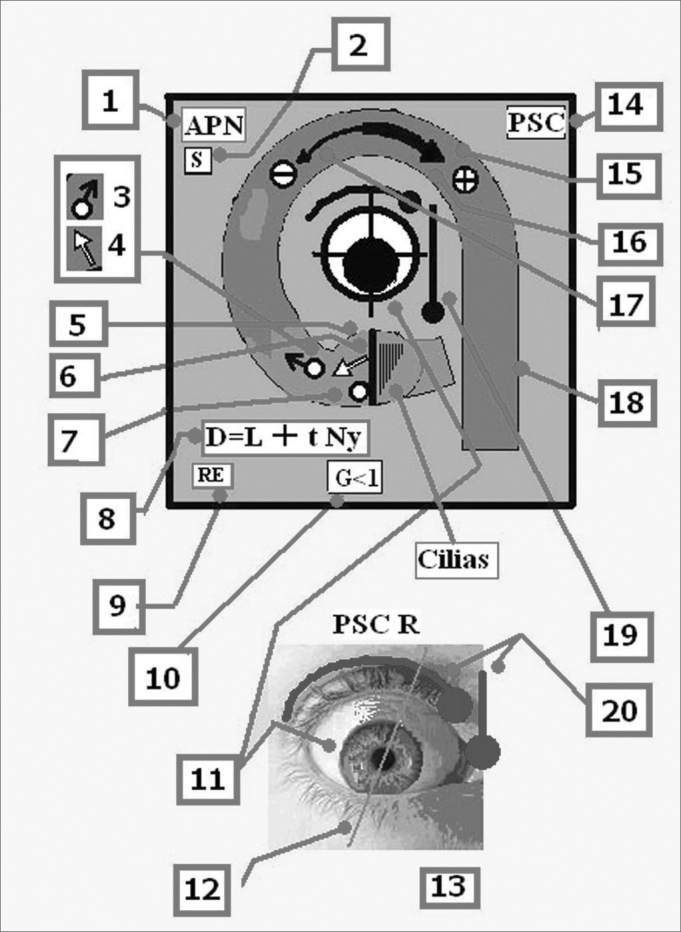
1-Acronym of the nerve, which innervates the semicircular canal. Ampullar Posterior Nerve. 2- Acronym of the saccule. The saccule shares its innervation with this semicircular canal. 3 -Initial Position of the otolith (t'0) 4- Direction of the tilt of the cupula 5 - Cupula. 6 - Crista.7 - Cupulolithiasis. 8 - Equation that estimates the total migration time of the otoconia crystals D: Duration, L: Latency, tNy: Time of duration of the nystagmus 9 - Acronym for the name which corresponds to the ear 10 - Gain of the semicircular canal. 11 - Position the eye adopts alter stimulating the canal. 12 - Vertical axis of the eye. 13 - Right ear. 14 - Name of the semicircular canal. 15 - Direction of the endolymph. 16 - The thick arrow shows that the excitatory stimulation is stronger than inhibition. 17 - The thin arrow shows the inhibitory stimulus. The inhibitory stimulus is ampullipetal. 18 - Each semicircular canal is color coded, and those which share the same color work together when stimulated. 19 - Slow phases. 20 - The round headed indicates that movement of the eye, and they are placed to remember that during the Ny, the rotatory motion is clearer seen when the patient look outward, and vertical motion when he look inward.

**Figure 4:**
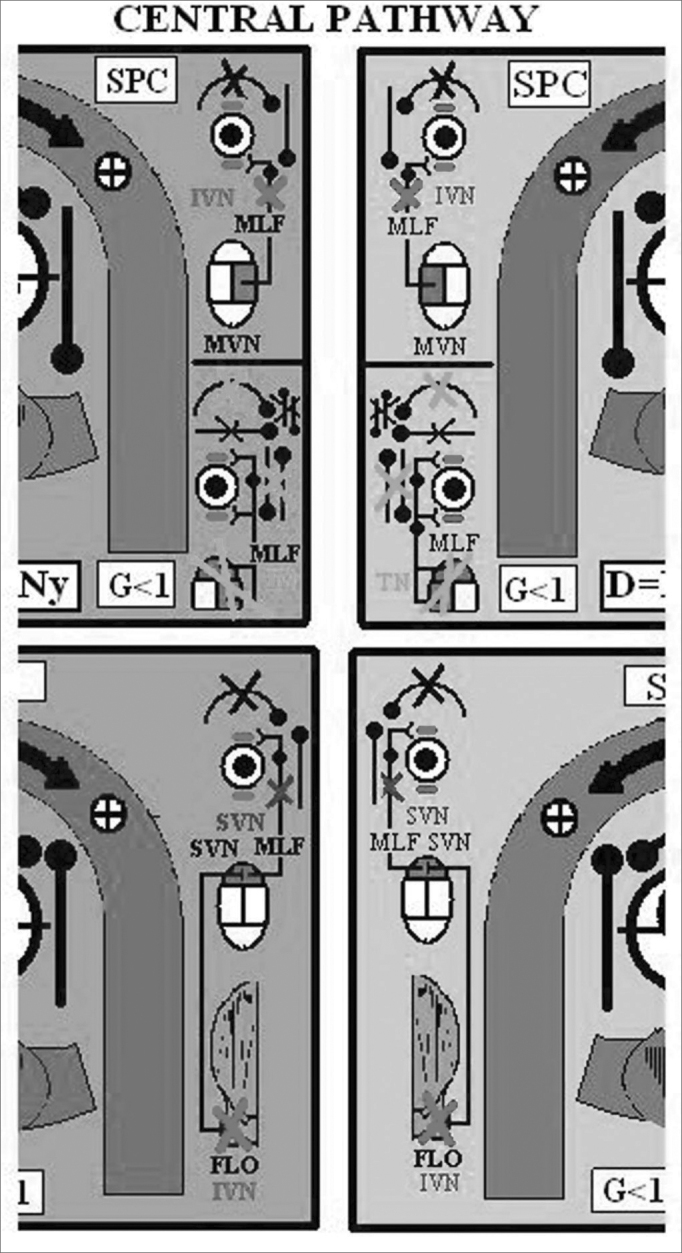
Central Pathway - Acronyms of figure 3: FLO: Cerebellar Flocculus, SVN: Superior Vestibular Nuclei, MVN: Medial Vestibular Nuclei, MLF: Medial Longitudinal Fasciculus, UBN: Up Beat Nystagmus (violet), NVI: Down Beat Nystagmus (red), NT: Torsional Nystagmus (green).

Each one of the faces of the SSCC has the following information as displayed in Fig. 3, in which the Right Posterior Semicircular Canal (RPSC) is shown as an example, since it is in this conduct where BPPV can often be found. Each one face presents the same information in the same location. The faces of the horizontal canals are arranged differently, however, with the same concept. A partial diagram of the central symbology is displayed in Fig. 4; however it is explained in greater detail in the users guide that accompanies the model. In the inferior faces of the horizontal SSCC there is a diagram of the most frequent vascular supplies to the inner ear, its innervation and vascular variations.

**Figure 5:**
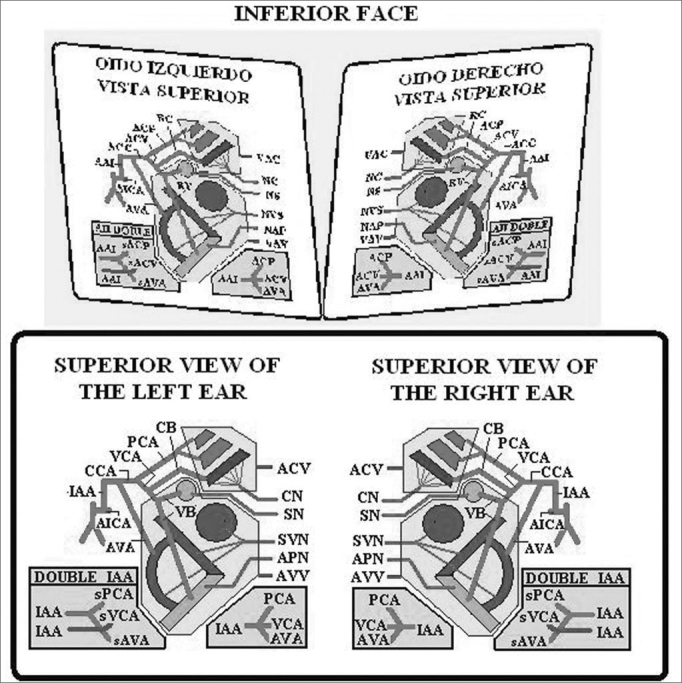
Inferior Face

**Figure 6:**
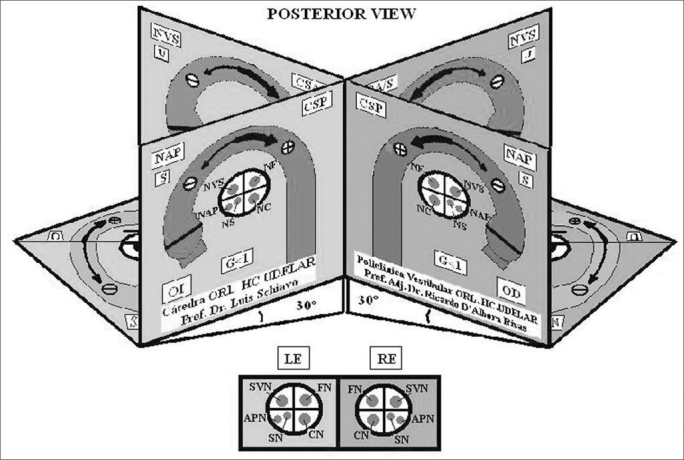
Posterior Face - Acronyms of Figure 1, Figure 2, Figure 4 and 5: A/SSC: Anterior/Superior Semicircular Canal, PSC: Posterior Semicircular Canal, H/LSC: Horizontal/Lateral Semicircular Canal, SVN: Superior Vestibular Nerve, APN: Posterior Ampullar Nerve, SN: Sacular Nerve, FN/VII: Facial Nerve. CN: Cochlear Nerve, U: Utricle. It shares its innervation and vascular supply with this canal, S: Saccule. It shares its vascular supply and innervation with this canal (in this particular case the Sacular Nerve) G: Gain: Ratio between the angular movement of the head and angular movement of the eye. D: Duration/Total length of otoconial migration. L: Latency, which is the time from the final position of the diagnostic Dix-Hallpike maneuver until the appearance of the first nystagmic beat. tNy: Corresponds to the total time of duration of nystagmus, RE: Right Ear, LE: Left Ear, AICA: Anterior Inferior Cerebellar Artery, IAA: Inner Auditory Artery, AVA: Anterior Vestibular Artery, CCA: Common Cochlear Artery, PCA: Posterior Cochlear Artery, CVA: Cochlear Vestibular Artery, CB: Cochlear Branch, RV: Vestibular Branch, sPCA: Similar to the Posterior Cochlear Artery, sCVA: Similar to the Cochlear Vestibular Artery, sAVA, Similar to the Anterior Vestibular Artery. ACV: Aqueduct Cochlear Vein, AVV: Aqueduct Vestibular Vein.

Following a color code, the arteries are in red, the veins in dark blue, and the venous drainage of the corresponding vein in light blue. The nerves are in green. Fig. 5. In the posterior face of the SSCC there is a diagram that shows the distribution and relation of the different nerves that pass by the internal auditory canal (posterior sight) Fig. 6. The model has been very well received at various Latin American scientific conferences as listed below.

• Primer Curso Internacional de Neurotología. Centro Neurológico ABC. México DF. 1315/10/2008.
• 6° congreso Nacional de ORL y XVII encuentro de ORL del Interior de la República. Hotel Jean Clevers. Punta del Este. Maldonado. Uruguay 31/10 y 1 y 2/12 2008.
• Seminario de Interacoustics LimaPerú 13/12/2008.
• VI Simposio INEBA de NeuroOtología 11 de Junio de 2009. BsAs Argentina
• 71° Jornadas Rioplatenses de ORL 1416 de Julio de 2010, as a Conference.

In addition, this model is used by the ENT Service of the Hospital de Clínicas, Facultad de

Medicina, Universidad de la República Oriental del Uruguay. The model is often understood after only verbal instruction; however, a print user manual is included.

## DISCUSSION

This 3D model is a useful tool to teach concepts of anatomy, physiology and pathology without the need of texts. It is a practical, low cost tool for use in private and academic settings.

This essay has been abridged and we advise reading the complete user manual for a more complete understanding of this tool.

## CONCLUSION

This model is a practical, low cost tool for use in private and academic settings. It requires only basic prior knowledge and is easy to learn.
